# Supplementary material for: Patterns of Intron Gain and Loss in Fungi
Source: PLoS Biol. 2004 Nov 30;2(12):e422. doi: 10.1371/journal.pbio.0020422 (PMC532390; doi:10.1371/journal.pbio.0020422)
Supplement: Table S1 — Also available at http://genes.mit.edu/NielsenEtAl/. (4.3 MB ZIP). [file pbio.0020422.st001.zip › NielsenEtAl/html/768.html]

AN4231.1.NCU07456.1.MG04586.1.FG01131.1


```
 CLUSTAL W (1.82) Multiple Sequence Alignments - Introns Inserted


Sequence 1: MG04586.1	214 aa
Sequence 2: FG01131.1	234 aa
Sequence 3: NCU07456.1	255 aa
Sequence 4: AN4231.1	234 aa
Alignment Length: 260 aa
Number Identitical Residues: 98 aa
Alignment Score (without introns) 4904


MG04586.1 	MFTGIVEEIGV1VAELNPHD--ATGGTSLTISLP-------TGSSLLSDCHDGDSIAVNG
NCU07456.1	MFTGIVEEIGE1VTTYTPNDPLSKNGTTLTITLLPSSSSSSSSSGLLSDCHLGDSIAVNG
FG01131.1 	MFTGIVEEVGV1VSKLDNND--STGGTSLTISIP-------SGSQLLSDCHDGDSISVNG
AN4231.1  	MFTGLVETIGT1VSSLEPLDTSASGGGGTSLTIT-------DCDTILTDVQLGDSISVNG
          	****:** :*  *:     *. :..*   ::::          . :*:* : ****:***

MG04586.1 	~VCLTVTSFTPT---QFTVGVAPETLRVTDLGDLVKDSRVNLERAVRADTRMGGHFVQGH
NCU07456.1	~VCLTVTSFTLTPSASFTVGIAPETLRLTNLGSLRSGSRVNLERAVRADTRMGGHFVQGH
FG01131.1 	~VCLTVTSFTPE---AFTIGVAPETLRITNLGDLKENSNVNLERAVRADTRMGGHFVQGH
AN4231.1  	1ACLTVTAFEKN---WFKVGVAPETLRRTNLGSLTETSKVNLERAVKGETRMGGHIVQGH
          	 .*****:*       *.:*:****** *:**.* . *.*******:.:******:****

MG04586.1 	VDTTATIADKQADGNAVTMRFKPREGSDVLKYIVRKGYVALDGTSLTVTKVDD-AAGWWE
NCU07456.1	VDTVAEIIKITPDGEALTFRFRPKN-KEVLRYVVYKGFIALDGTSLTVTAVND-EEEWWE
FG01131.1 	VDTTAKILSVTKDGNALTFRFQPAR-RDMLRYIVYKGYIAIDGTSLTVTKVND-QEGWWE
AN4231.1  	VDTIAKIIAMTPDENSLVLRLQPRD-PAVLRYIVEKGFITLDGASLTITQVKDGEDGWFE
          	*** * *     * :::.:*::*     :*:*:* **::::**:***:* *.*.   *:*

MG04586.1 	VMLIVYTQERVVLAQKNVGDTVNVEVDVLAKYAEKSMAGYLSSLNKSDA-----------
NCU07456.1	VMLIAYTQERVVVAGKKVGETVNVEVDMTAKYVEKSLRGYLEGLLGSGSAGEQGQGEEKK
FG01131.1 	VMLIAYTQERVVVAQKKEGDTVNLEVDMMAKYAEKSLGGVVGSEPGSVASFP--------
AN4231.1  	IMLIAYTQEKIVTAAKRVGDFVNVEIDVVAKYVEKGVQAYFAGTAGGN------------
          	:***.****::* * *. *: **:*:*: ***.**.: . . .   .             

MG04586.1 	----------------------
NCU07456.1	KGRGLLEKVVEGIVKQGFS---
FG01131.1 	----FIEKIVERIVAQGFGAKQ
AN4231.1  	--MGILEKMVGRIVDEKLNGR-
          	   .  ..       .  ..
```
